# Using Artificial Intelligence to discriminate among PPA subtypes from a real‐world language sample

**DOI:** 10.1002/alz70858_105237

**Published:** 2025-12-26

**Authors:** Gent Celaj, Neguine Rezaii, Daisy Hochberg, Brad C. Dickerson, Bonnie Wong

**Affiliations:** ^1^ Frontotemporal Disorders Unit and Massachusetts Alzheimer's Disease Research Center, Department of Neurology, Massachusetts General Hospital and Harvard Medical School, Boston, MA, USA; ^2^ Department of Psychiatry, Massachusetts General Hospital, Boston, MA, USA

## Abstract

**Background:**

Advancements in artificial intelligence and natural language processing (NLP) have made it a valuable tool for diagnostic and clinical applications. Building on Rezaii et al. (2024), who used AI‐driven NLP tools to identify linguistic features discriminating Primary Progressive Aphasia (PPA) subtypes from a picture description task, the current study aimed to replicate and extend these findings using an ecologically‐valid language sample often derived from the clinical question, “What did you do for work?” (WDYDFW?). We hypothesized that features of sentence length, word frequency, and content word usage, would discriminate among PPA subtypes.

**Method:**

Speech samples from the WDYDFW? prompt were collected from 58 PPA participants (Mean age = 69.62 (SD = 7.38); 50.0% female) and 16 healthy controls (Mean age = 65.56 (SD = 4.95); 62.5% female). Digital language markers were extracted from transcribed samples using Quantitext, an NLP‐based toolbox our team has developed. Sentence length (average # words per sentence), word frequency (commonality in the English language of words used), content word usage (words that provide meaning, i.e., nouns, verbs, adjectives, adverbs), and syntax frequency (simple vs complex sentences structure) previously identified as key distinguishing linguistic features (identified by Rezaii et al. as key discriminating language features) were quantified for each sample. One‐way ANOVA with cohort as the dependent variable and linguistic features as the independent variables was conducted to detect differences in language markers among groups.

**Result:**

Preliminary analyses indicated individuals with nfvPPA produced significantly shorter sentences (F(3,70) = 12.583, *p* < .001) and lower‐frequency words compared to controls (F(3,70) =17.395, *p* = .01,), lvPPA (*p* < .001), and svPPA (*p* < 0.001). Controls used lower frequency words than lvPPA (F(3,70) =17.395, *p* = 0.02). lvPPA participants produced fewer content words than nfvPPA (F(3,70) = 3.77, *p* < 0.025) and control individuals (*p* < 0.05). Syntax frequency did not differ across groups.

**Conclusion:**

Consistent with previous findings, NLP has the potential to discriminate among PPA subtypes using unstructured language samples. This study underscores the potential of AI tools to classify atypical cognitive‐behavioral syndromes. Analyses are ongoing to assess impact of symptom severity (CDR) and to validate findings against structured language samples.

